# Sodium channel β2 subunit promotes filopodia-like processes and expansion of the dendritic tree in developing rat hippocampal neurons

**DOI:** 10.3389/fncel.2013.00002

**Published:** 2013-01-25

**Authors:** Marta Maschietto, Stefano Girardi, Marco Dal Maschio, Michele Scorzeto, Stefano Vassanelli

**Affiliations:** Department of Biomedical Sciences, University of PadovaPadova, Italy

**Keywords:** sodium channel, β2 subunit, filopodia, rat hippocampal neuron, CHO-K1

## Abstract

The β2 auxiliary subunit of voltage-gated sodium channels (VGSCs) appears at early stages of brain development. It is abundantly expressed in the mammalian central nervous system where it forms complexes with different channel isoforms, including Na_v_1.2. From the structural point of view, β2 is a transmembrane protein: at its extracellular N-terminus an Ig-like type C2 domain mediates the binding to the pore-forming alpha subunit with disulfide bonds and the interactions with the extracellular matrix. Given this structural versatility, β2 has been suggested to play multiple functions ranging from channel targeting to the plasma membrane and gating modulation to control of cell adhesion. We report that, when expressed in Chinese Hamster Ovary cells CHO-K1, the subunit accumulates at the perimetral region of adhesion and particularly in large lamellipodia-like membrane processes where it induces formation of filopodia-like structures. When overexpressed in developing embryonic rat hippocampal neurons *in vitro*, β2 specifically promotes formation of filopodia-like processes in dendrites leading to expansion of the arborization tree, while axonal branching remains unaltered. In contrast to this striking and highly specific effect on dendritic morphology, the targeting of functional sodium channels to the plasma membrane, including the preferential localization of Na_v_1.2 at the axon, and their gating properties are only minimally affected. From these and previously reported observations it is suggested that β2, among its multiple functions, may contribute to promote dendritic outgrowth and to regulate neuronal wiring at specific stages of neuronal development.

## Introduction

Voltage-gated sodium channels (VGSCs) (Goldin et al., 2000; Catterall et al., 2005) are fundamental for neuronal excitability and action potential propagation. They are widely expressed within the nervous system as heteromeric transmembrane complexes of a pore-forming α subunit, existing in nine isoforms (Na_v_1.1–Na_v_1.9), and at least one auxiliary β subunit. Four β subunits (β1–β4) have been identified (Catterall, 2000; Catterall et al., 2005): β1 and β3 are non-covalently linked to the α subunit, while β2 and β4 are disulfide-linked to α by means of their N-terminal Ig-like domain (Hartshorne et al., 1982; Morgan et al., 2000; Yu et al., 2003; Chen et al., 2012). Their C-terminus, instead, is a short 34 aminoacids region. β subunits are seen as potential candidates to play multiple functions including shifting sodium channels voltage-dependence and regulating their surface expression or promoting cell-extracellular matrix and cell-cell adhesion interactions. Overall, they are emerging as modulators of cell excitability and adhesive properties encompassing a wide spectrum of actions within and outside the nervous system (Kaczmarek, 2006; Brackenbury and Isom, 2008; Brackenbury et al., 2008b; Patino and Isom, 2010).

The β2 subunit makes no exception. Abundantly expressed in the rat brain, it is found associated with several Na_v_ isoforms (Catterall et al., 2005) and following various time- and region-dependent patterns during development (Isom et al., 1995; Gastaldi et al., 1998; Gong et al., 1999; Chen et al., 2002). Regulating cell adhesion and migration-related events (Kaczmarek, 2006; Brackenbury et al., 2008b; Patino and Isom, 2010) and axonal growth (Srinivasan et al., 1998; Xiao et al., 1999) has been considered among its putative roles. Particularly, as a physiological substrate of β-site APP cleaving enzyme 1 (BACE1) and γ-secretase (Kim et al., 2005; Wong et al., 2005), two proteolytic enzymes central to Alzheimer's disease pathogenesis (Gandy, 2005), β2 was found to affect adhesion/migration in CHO-K1 cells, besides regulating Na_v_1.1 mRNA and protein expression levels (Kim et al., 2005, 2007). Intriguingly, β2 has been shown to heavily affect membrane morphology. *Xenopus* oocytes expressing β2 were characterized by an increment of microvilli extension (Isom et al., 1995). Similarly, an increase of filopodia-like processes was induced in HEK293 cells (Zimmer et al., 2002). It is still unclear whether related functions could be relevant for neurons. On the other end, as expected for a channel auxiliary subunit, β2 was shown to modulate Na_v_ membrane expression and targeting (Malhotra et al., 2000; Pertin et al., 2005; Lopez-Santiago et al., 2006; O'Malley et al., 2009) and to shift Na_v_ voltage-dependence of activation and inactivation. This latter effect appears to vary by cell type or stage of development in terms of extent and shifting direction (Isom et al., 1995; Qu et al., 2001; Chen et al., 2002; Yu et al., 2003).

In conclusion, β2 has the potential to play variegated functions characterized by a channel modulator/adhesion molecule dualism. Certainly, the abundant expression in the mammalian central nervous system, including embryonic stages, supports the hypothesis that it plays a physiological role also during neuronal development. To deepen understanding of β2 adhesion-related functions, particularly during development, we investigated the effect of β2 expression first in CHO-K1 cells and then in developing rat hippocampal neurons. By analyzing changes in cell morphology and the subcellular distribution of a newly synthesized β2 fluorescent chimera with a combination of Total Internal Reflection Fluorescence (TIRF) and epifluorescence microscopy, we show that β2 can act as adhesion molecule promoting outgrowth of filopodia-like processes and specifically enhancing branching of the dendritic tree in developing neurons.

## Materials and methods

### Plasmids and cloning strategies

A β2-ECFP chimera (subsequently named β2C) was created by linking ECFP to the C-terminus of the rat brain β2 subunit. The cDNA coding for the rat β2 precursor (with N-terminal signal peptide) was amplified from a donor vector (kindly given by Prof. A. L. Goldin, Department of Microbiology and Molecular Genetics, Department of Anatomy and Neurobiology, University of California, Irvine, CA, USA) with 5′ NheI and 3′ ApaI ends (forward primer: 5′cggGCTAGCcatgcacagggatgcctggct3′; reverse primer: 5′atcGGGCCCgcgcttggcgccatctt3′), restricted with specific enzymes (Promega, Italy) and cloned into linearized pcDNA3.1(–) (Invitrogen, Italy). The ECFP cDNA was amplified by PCR from a donor vector (kind gift by Prof. T. Pozzan, Department of Biomedical Sciences, University of Padova) with 5′ EcoRI and 3′ BamHI ends (forward primer: 5′ccGAATTCatggtgagcaagggc3′; reverse primer: 5′ggGGATCCggctacttgtacagctcg3′; the ends are in capital letters, the stop codon is underlined), and subcloned in frame and downstream of β2. The linker between β2 and ECFP was the amino acid sequence GPSRLERPPLCWISAEF.

β2 cDNA was amplified from donor vector with 5′ EcoRI and 3′ BamHI ends (forward primer: 5′agGAATTCcatgcacagggatgcctggct3′; reverse primer: 5′cgcGGATCCgcgttacttggcgccatcttc3′) and subcloned into pcDNA3.1(–) to express the subunit in mammalian cells without fluorescence at its C-terminus. EcoRI/BamHI amplified ECFP cDNA was subcloned alone into pcDNA3.1(–) for control transfections. All custom single strand oligonucleotides for PCRs (Sigma-Aldrich, Italy) were designed on GenBank cDNA sequences by means of Primer3 software (http://primer3.sourceforge.net/). PCR reactions were performed using alternatively Expand™ High Fidelity Taq (Roche) or Platinum® Taq DNA Polymerase (Invitrogen), according to the manufacturer's instructions. Cloning was verified by standard sequencing (BMR Genomics, Italy).

The rat brain Na_v_1.2 α subunit rIIA isoform (subsequently named Na_v_1.2 or α; Auld et al., 1988), cloned into the mammalian expression vector pCDM8, was a kind gift of Prof. W. A. Catterall (Department of Pharmacology, University of Washington).

### Cell cultures and transfections

CHO-K1 (ATCC, USA) were maintained in F-12 Nutrient Mixture—Ham—supplemented with 10% (v/v) heat-inactivated FBS, 10 u/ml penicillin and 10 μg/ml streptomycin, in a humidified atmosphere at constant temperature (37°C) and CO_2_ concentration (5% v/v). For transfection experiments, 17 × 10^3^ cells/cm^2^ cells were plated onto glass coverslips; 1 μg for each plasmid, previously amplified and purified from bacterial strains (HiSpeed Plasmid Maxi Kit, QIAGEN, Italy), was transfected with Arrest-In™ (Open Biosystems, Celbio, Italy) two days after plating.

Wistar rats (Charles River Laboratories, USA) were maintained in the Animal Research Facility of the Department of Biomedical Sciences (University of Padova) under standard environmental conditions. All the experiments dealing with animals have been approved by the Institutional Ethical Committee (CEASA) of the University of Padova. Primary neurons were dissociated with papain from freshly dissected E18 rat embryos hippocampi (E1 being the day when vaginal plug was found; Berry, 1970). Glia was reduced by pre-plating, and hippocampal neurons were suspended in DMEM with GlutaMAX-1, supplemented with 10% FBS, 1 u/ml penicillin, and 1 μg/ml streptomycin. About 34 × 10^3^ neurons/cm^2^ were plated onto glass coverslips (previously coated with 10 μg/ml poly-L-lysine) in L15 medium supplemented with 5% FBS and incubated at 37°C and 5% CO_2_ for 90 min. L15 was then substituted with Neurobasal™ Medium added with 1% FBS, 1% GlutaMAX™-1, and 2% B-27. Three days after dissociation, neurons were maintained in Neurobasal™ Medium supplemented with 2% B-27 (Brewer, 1997; Vassanelli and Fromherz, 1997). One microgram of each plasmid was transfected into 6 days *in vitro* (DIV) neurons by means of Lipofectamine™ 2000. CHO-K1 and neurons culture and transfection reagents, if not otherwise indicated, were purchased from Invitrogen.

### Cells fixation and labeling

One day after transfection, CHO-K1 and hippocampal neurons were fixed with 10% formaline (Sigma-Aldrich) and washed with PBS, pH 7.4 (in mM: 137.0 NaCl, 2.7 KCl, 10.0 Na_2_HPO_4_, 2.0 KH_2_PO_4_). For long-term transfections of neurons, cells were fixed 9 days after transfection (i.e., at 15 DIV). For antibody labeling, cells were permeabilized with 0.1% Triton X-100 (Sigma-Aldrich) in PBS at room temperature for 20 min, washed with 0.1% Tween-20 (Sigma-Aldrich) in PBS, and incubated for 30 min with 1% BSA (Sigma-Aldrich) in PBS. A primary monoclonal antibody against the Na_v_1.2 α subunit intracellular C-terminus (Research Diagnostic, Fitzgerald, GA, USA) was added overnight at 4°C (2.0 μg/ml) and visualized by using 1:300 anti-mouse IgG Alexa Fluor 568 (Invitrogen) after washing with 0.1% Tween-20 and incubating with 1% BSA. Neuronal endogenous β2 and wild-type subunit expressed in CHO-K1 were immunolabeled with 2 μg/ml polyclonal primary antibody (ASC-007, Alomone Labs, Israel) with intracellular C-terminal epitope (Kaplan et al., 2001) and 1:300 anti-rabbit IgG Alexa Fluor 514 (Invitrogen). Neuronal MAP2 protein was stained with polyclonal primary antibody (PRB-547C, Covance) and 1:300 anti-rabbit IgG Alexa Fluor 488 (Invitrogen). Neurofilaments H were immunolabeled with primary monoclonal antibody (SMI-31R, Covance) and 1:300 anti-mouse IgG Alexa Fluor 488 (Invitrogen). Actin was labeled with Alexa Fluor 568 phalloidin (Invitrogen). For FM staining, non-fixed cells were visualized after 1 min staining at 4°C with 5 μg/ml FM® 1−43 Lipophilic Styryl Dye in HBSS (Invitrogen) and for a maximum observation time of 5 min (to avoid artifacts caused by dye internalization).

### TIRF microscopy and image analysis

Epifluorescence and TIRF microscopy (Axelrod, 1981, 2001) was performed using a commercial White-Light TIRF™ apparatus mounted on a Nikon TE2000E stage and a CFl Plan Apochromat TIRF 60x/1.45 oil objective with 80–200 nm penetration depth (Nikon Instruments, England). Emission bands: 460–500 nm for ECFP, 520–550 nm for Alexa Fluor 488, 515–555 nm for Alexa Fluor 514, and 578–632 nm for Alexa Fluor 568 and phalloidin. Images were analyzed with the ImageJ (http://rsbweb.nih.gov/ij/) and Image-Pro Plus 6.0 (MediaCybernetics, USA) softwares.

#### Subunits localization and expression

***Intensity difference analysis.*** A semi-quantitative estimate of β2C expression in the plasma membrane was obtained from TIRF images by comparing β2C and FM1-43 fluorescence (hereafter indicated as *F*_β_ and *F*_*M*_, respectively). This intensity difference analysis procedure was previously proposed to analyze membrane expression of a genetically targeted calcium sensor (Shigetomi et al., 2011). Briefly, the analysis was performed through normalization and then subtraction of *F*_*M*_ from *F*_β_, under the assumption that the distribution of FM1-43 was homogenous in the plasma membrane. In this way, after subtraction from *F*_β_of signal components due to reduced membrane-to-substrate distance or excitation of non-adherent membranes by the evanescent wave, fluorescence intensity was directly related to the level of β2C membrane expression. For each cell, a region of interest (ROI) of 5 × 5 μm^2^ area with approximately homogeneous β2C and FM1-43 fluorescence was selected in the center of the cell and average intensities, < *F*_β_ > and < *F*_*M*_ >, computed. After normalization of the FM1-43 image to < *F*_β_ >, FM1-43 intensities were subtracted from the β2C image and the result plotted in pseudo-color code.

***Radial β2C expression profiles.*** The β2C fluorescence radial profiles in the plasma membrane of CHO-K1 cells were measured in TIRF images along manually selected linear segments (10 μm length) crossing the cell's edge and then compared to FM1-43 signals. When selecting segments, care was taken to exclude lamellipodia-like structures. A border-to-center ratio was computed for each selection from a five pixels average at the border and center regions of the cell.

***α and β2C co-expressing cells.*** In α and β2C co-expression studies, the expression level of Na_v_1.2 α subunit in the plasma membrane was estimated from TIRF images of immunolabeled cells. Two parameters were extracted: a background noise, *N*, and a signal value, *S*. *N* was defined as the average fluorescence intensity of 10 ROIs (3 × 3 μm^2^ each) in non-transfected cells; *S* was the average intensity of 10 ROIs in the transfected cell. Na_v_1.2 expression was then reported in arbitrary units (AU) and represented the signal-to-noise ratio *R* = (*S* − *N*)/*N*. An identical signal processing criterion was used for evaluation of β2C expression in these experiments.

***Cellular processes.*** Lengths of filopodia-like processes were measured with ImageJ software in CHO-K1 cells expressing β2C or co-expressing α and β2C and compared with mock-transfected cells stained with phalloidin. Processes were then divided in 3 groups (with length <4 μm, 4/8 μm, >8 μm) and frequency reported as percentage. In neurons, filopodia-like structures were counted manually on a 20 μm long arbitrary selection of the dendritic tree of each neuron. A quantitative estimate of neuronal arborization was obtained by defining a branching area index as the ratio between the area occupied by branches and the total imaged area (excluding somatas). For this analysis, raw images were acquired in standard epifluorescence by a 2 Mpixel CCD camera DS-2MBWc (Nikon Instruments) and subsequently filtered with a HighGauss and Median filter.

### Whole-cell voltage-clamp

Whole-cell patch-clamp recordings were performed 24 h after transfection using an Axopatch 200B amplifier (Molecular Devices, USA). The voltage protocol for steady-state activation consisted of test pulses of 60 ms duration with increasing amplitude (5 mV step) starting from a –90 mV holding potential. Current transients due to passive RC pipette-membrane components and leakage were subtracted using a P/4 protocol. The membrane sodium conductance was computed from the applied voltage, corrected for the access resistance to obtain the true intracellular voltage, the corresponding whole-cell current and the measured sodium reversal potential. The sodium conductance steady-state activation curve was fitted, after normalization of the conductance to its maximum value, by a Boltzmann distribution describing steady-state activation in terms of the channel gating charge, *q*_*g*_, and gating voltage, *V*_*g*_ (Fry et al., 2003). The specific conductance was calculated by normalizing the whole-cell sodium conductance to the membrane capacitance (which was determined offline from the RC current transients recorded during P/4 pulses through fitting by exponential functions). Pyramidal-like neurons were selected on the basis of morphological criteria for recording. Extracellular and intracellular solutions used for voltage-clamp records were, respectively (in mM): 135.0 NaCl, 5.4 KCl, 1.0 MgCl_2_, 1.8 CaCl_2_, 10.0 glucose, 5.0 HEPES (adjusted to pH 7.4 with 1 N NaOH); 140.0 KCl, 2.0 MgCl_2_, 5.0 EGTA, 5.0 HEPES (adjusted to pH 7.3 with 1 N KOH). Potassium channels blockers (20 mM TEA and 200 μ M 4-AP; Sigma-Aldrich) were included in the pipette solution. Neurons showing residual A-type fast inactivating potassium currents were not considered. Pipettes resistances were 2–3 MΩ in extracellular solution. Recordings showing non-negligible space-clamp artifacts and series access resistances larger than 15 MΩ were discarded from analysis.

### Statistical analysis

Statistical analysis and fitting was done using GraphPad Prism (GraphPad Software). Comparisons between data groups were performed using Student's *t*-tests and expressed as mean ± SEM, with *p* < 0.05 as criterion for significance. Fitting of the sodium channel activation curve by a Boltzman distribution was obtained by non-linear regression and forcing the relative conductance (*G*_*M*_/*G*_0_) to one in correspondence of the highest depolarizing voltage.

## Results

### β2 accumulates at the periphery and in membrane processes of CHO-K1 cells

**Figure 1:**
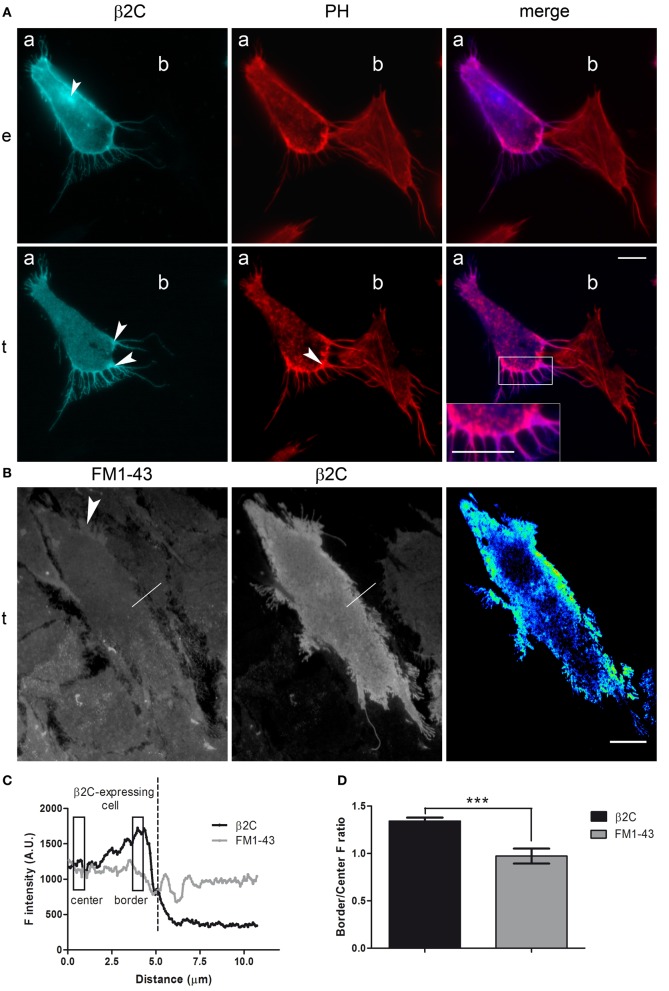
**β2C distribution pattern in CHO-K1 cells. (A)** Fluorescence microscopy micrograph (epifluorescence, *e*, and TIRF, *t*) showing a representative CHO-K1 cell expressing β2C **(*a*)** and a non-expressing cell **(*b*)** in its proximity. Phalloidin staining of F-actin in the two cells (PH) is visible together with the merged image of β2C and PH channels. β2C channel: white arrows point in *e* to a perinuclear region of subunit retention and in *t* to lamellipodia- and filopodia-like structures with enhanced fluorescence. PH channel: the white arrow indicates F-actin accumulation in membrane processes of cell **(*a*)**. Merge: note the blow-up showing correspondence between F-actin and β2C localization. **(B)** TIRF image with FM1-43 stained cells and some of them expressing β2C (*left*, FM1-43 channel; m*iddle*, β2C channel). The white arrow in the FM1-43 channel points to a typical lamellipodia-like process with higher fluorescence. Superimposed to both FM1-43 and β2C channels is one 10 μm long linear selection crossing the cell edge (*white segment*) whose fluorescence intensity profile is plotted in **(C)**. (*right*) Pseudo-color image obtained by intensity difference analysis of the cell in the center (signal intensity increases from cold to warm colors). Here, the higher intensity in lamellipodia-like processes and at the cell periphery suggests β2C accumulation in these regions. Scale bars **(A,B)**: 10 μm. **(C)** Fluorescence intensity profiles of β2C (*black*) and FM1-43 (*gray*) signals along the linear selection shown in **(B)**. FM1-43 and β2C signal baselines were juxtaposed for easier comparison. β2C fluorescence rose from center to periphery reaching a maximum at the cell border, thus dropping in correspondence of the cell edge. FM1-43 fluorescence was relatively constant until the cell edge, and then it rippled because the selection was intercepting a neighboring FM-43 stained cell (not expressing β2C). **(D)** Comparison of β2C and FM1-43 border-to-center ratios. Representative center and border regions considered for analysis are indicated by rectangles in **(C)**. For each out of 8 different cells, the average of ratios in 10 selections was considered for comparison confirming a preferential localization of β2C at the cell border (β2C: 1.34 ± 0.11; FM1-43: 0.97 ± 0.08, *n* = 8, ^***^p < 0.0001. Data expressed as mean ± SEM).

β2 expression was firstly investigated in CHO-K1 cells. Indeed, as native sodium channels are nearly absent in this cell line (Lalik et al., 1993), the system was offering the opportunity to study β2 in a context where it is not involved in the formation of channel complexes. A β2-ECFP chimera (β2C) was engineered (see Materials and Methods) to observe the subunit subcellular distribution by fluorescence microscopy at high spatial resolution. A representative cell expressing β2C is shown in Figure 1A as it appears under epifluorescence and TIRF microscopy (panel *e* and *t*). Overall, the subunit displays its normal preferential localization to the plasma membrane (Isom et al., 1995) with the exception of a few intracellular perinuclear spots, visible in epifluorescence, that were likely caused by accumulation in intracellular membranes as already reported by Zimmer et al. (2002) in HEK293 cells. In the TIRF image, a higher β2C fluorescence intensity is found at the cell periphery and, particularly, in membrane processes. As evidenced by phalloidin labeling of F-actin (PH), there was a good correspondence between these high fluorescence intensity regions and sites rich of actin filaments, typically indicating nascent adhesions and membrane protrusions (Parsons et al., 2010). To assess whether the increase of the β2C signal in these regions was indeed due to higher protein membrane expression and not to unrelated phenomena (i.e., reduced membrane-substrate distance or excitation of both adherent and non-adherent membrane by the evanescent wave), we compared β2C fluorescence with that of FM1-43, a dye which integrates and distributes homogeneously in the cell membrane (Figure 1B). FM1-43 fluorescence was homogeneous in the cell body but, similarly to β2C, also enhanced in correspondence of filopodia- and lamellipodia-like structures. We concluded that the higher β2C signal at the periphery of cell adhesion and in processes was due, at least in part, to tighter adhesion or spurious excitation of non-adherent membranes. Thus, the signal component directly related to a higher level of membrane protein expression was identified by first normalizing and then subtracting the FM1-43 fluorescence from the β2C channel through a quantitative intensity difference analysis (see Materials and Methods). The resulting image showing regions of β2C accumulation in a representative cell is visible in Figure 1B. For easy of visualization, intensities are displayed in pseudo-color. Prevalence of warm colors in lamellipodia- and filopodia-like processes indicates preferential localization of β2C in the adherent membrane of these structures. Overall, all observed β2C expressing cells were showing a similar pattern. Alternatively, to analyze the pattern of β2C distribution and particularly outside lamellipodia-like processes, fluorescence was measured along selections perpendicular to the membrane's edge (10 μm length) that were crossing the cell border (Figure 1B). β2C but not FM1-43 intensity profiles typically raised in correspondence of the cell periphery (Figure 1C) and averages of periphery-to-center ratios showed a significant 34% increase for β2C compared to FM1-43 (Figure 1D). Taken together, these results indicated that β2C was preferentially localized in membrane processes and, overall, at the periphery of the adherent membrane in CHO-K1, a pattern that is typically observed in case of proteins involved in cell adhesion (Parsons et al., 2010).

### Na_v_1.2 α subunit causes intracellular sequestration of β2 and reduces formation of membrane processes

**Figure 2:**
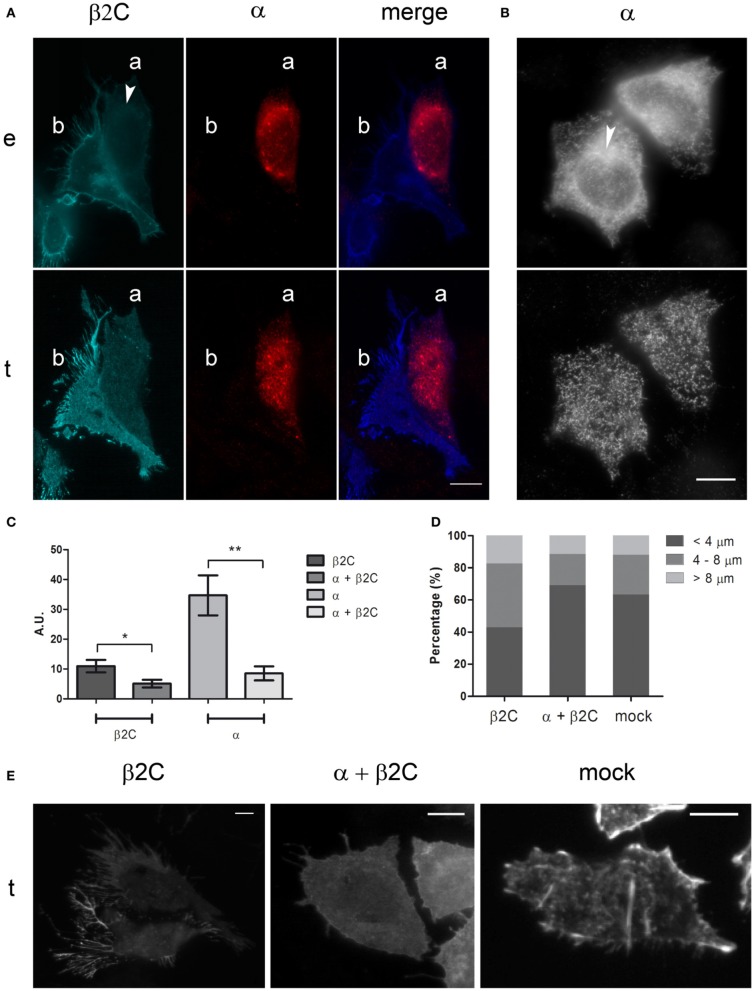
**Na_v_1.2 α subunit inhibits membrane processes formation by causing intracellular sequestration of β2C. (A)** Epifluorescence (*e*) and TIRF (*t*) micrographs of two representative cells co-expressing β2C and α (cell ***a***) or expressing β2C alone (cell ***b***). α was antibody labeled. From left to right: β2C channel (cyan), α channel (red), merged image. White arrow indicates β2C intracellular retention around the nucleus. Note the lower level of fluorescence in cell **(*a*)** with respect to **(*b*)** in the β2C channel and the intracellular co-localization in **(*a*)** of the two subunits in the merged image, hinting to α subunit as the cause of reduced membrane expression of β2C. Most notably, membrane processes appear to be reduced in **(*a*)** with respect to **(*b*)**. **(B)** Micrographs of α transfected cells (*e* and *t* as in **A**). Despite perinuclear retention (*arrow* in *e*), α subunit targeting to the plasmatic membrane remains significant (*t*). Scale bars **(A,B)**: 10 μm. **(C)** Averaged TIRF signals of cells expressing β2C or co-expressing α and β2C, normalized to basal noise and expressed in arbitrary units (AU). β2C channel: β2C cells (10.98 ± 2.07; *n* = 10), α + β2C cells (5.11 ± 1.29; *n* = 10). α channel: α cells (34.69 ± 6.70; *n* = 10), α + β2C cells (8.53 ± 2.35; *n* = 10). Data expressed as mean ± SEM. A significant decrease (^*^p = 0.0271) of β2C targeting to the plasmatic membrane is found when α is co-expressed. Similarly, a significant decrease (^**^p = 0.0017) of α targeting to the plasmatic membrane is observed in case of β2C co-expression. **(D)** Percentage of filopodia-like structures with length <4 μm, 4/8 μm, >8 μm as counted in 20 CHO-K1cells expressing β2C (*n* = 247), α and β2C (*n* = 184), or mock (*n* = 167) from at least three independent transfections. Note the increased percentage of filopodia-like processes with length >4 μm in β2C expressing cells. **(E)** Representative cells expressing β2C and α + β2C (β2C channel) and mock (phalloidin staining) from the populations analyzed in **(D)**. Scale bars: 10 μm.

**Figure 3:**
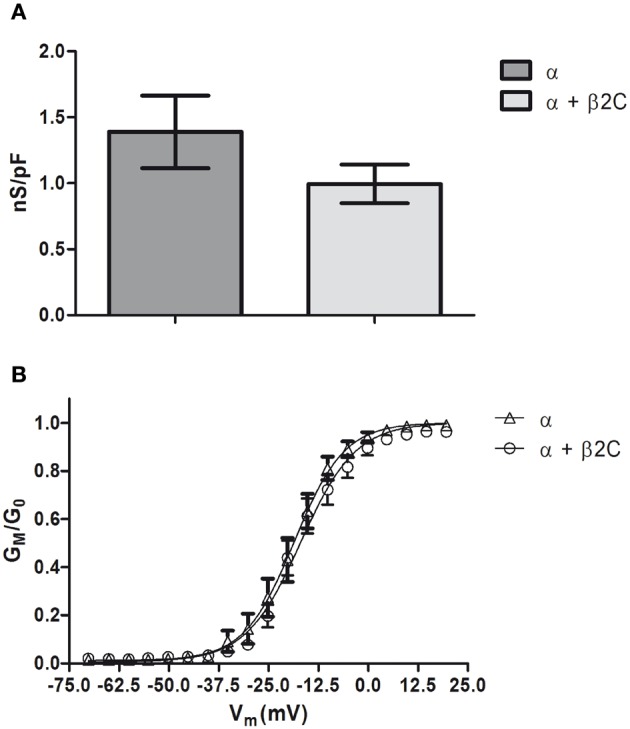
**Na_v_1.2 conductance in CHO-K1 cells is slightly affected by β2C co-expression. (A)** Whole-cell peak sodium conductance, normalized to membrane capacitance, as measured in CHO-K1 cells expressing Na_v_1.2 alone or co-expressing Na_v_1.2 and β2C 24 h after transfection. The voltage-clamp protocol consisted of a 60 ms test pulse to +10 mV starting from a −90 mV holding potential. A slight, albeit not significant (*p* = 0.2062), decrease of average conductance was observed in co-transfected cells (α = 1.39 ± 0.27 nS/pF, *n* = 15. α + β2C = 0.99 ± 0.14 nS/pF, *n* = 16). Data are expressed as mean ± SEM. **(B)** Voltage-dependence of sodium channels activation in the same α (*triangles*) and α + β2C (*circles*) neurons as in **(A)**. *G*_*M*_/*G*_0_ is the relative conductance (actual conductance divided by the maximum conductance) and *V*_*m*_ the intracellular voltage. Data were fitted by Boltzmann distributions (continuous lines) showing slight significant differences (*p* = 0.0051) between the two conditions in terms of half-voltage (β2C, *V*_0.5_= −18.56 ± 0.14 mV; α + β2C, −16.94 ± 0.53 mV) and slope (β2C, *s* = 6.29 ± 0.12 mV/e-fold conductance change; α + β2C, *s* = 6.89 ± 0.45 mV/e-fold).

Since β2 is recognized as an accessory subunit of VGSCs, its subcellular distribution and effect on CHO-K1 morphology were also investigated when co-expressed with the Na_v_1.2 main pore-forming α subunit. This Na_v_ isoform was demonstrated to associate with β2 in the rat hippocampus and other brain regions (Gong et al., 1999), thus representing a suitable physiological binding target of the accessory subunit. When co-expressed with Na_v_1.2 in this non-neuronal cell line, β2C was mainly retained in the perinuclear region, in contrast to the dominant membrane targeting that was observed when the subunit was expressed alone (Figures 2A,C). Considering the similar distribution pattern of α and β2C in the merged image of Figure 2A, we can speculate that Na_v_1.2 was causing β2C retention in endomembranes through formation of channel complexes lacking appropriate membrane targeting signals. A similar pattern was found when co-expressing α and the wild-type β2, thus excluding the possibility of a chimera-specific effect (not shown). The α subunit, when expressed alone, showed a partial accumulation in perinuclear structures, although maintaining a certain degree of membrane expression (Figures 2B,C). Statistical analysis over a population of co-expressing and non-co-expressing cells demonstrated a significant 2-fold decrease of the β2C signal in the plasma membrane in case of α—β2C co-expression. Similarly, a significant 4-fold decrease of the α subunit signal in the plasmatic membrane was found when α and β2C were co-expressed (Figure 2C). Interestingly, when analyzing the effect of co-expression on membrane processes, we found that the percentage of long (more than 4 μm) filopodia-like structures was significantly higher in β2C transfected cells with respect to α and β2C co-expression and mock conditions (Figures 2D,E). These observations indicated that β2C expression promotes outgrowth of filopodia-like cellular processes, a process that requires targeting of the subunit to the membrane. To investigate the effect of β2C expression on functional sodium channels, sodium currents were measured in CHO-K1 cells co-transfected with the α and β2C subunits and compared to cells expressing α alone. Overall, a tendency, albeit not significant, to a decrease of specific sodium conductance was observed in α and β2C co-expressing cells (Figure 3A). Apparently, despite reducing the membrane targeting of the α subunit (Figure 2C), β2C expression did not significantly affect the membrane targeting of mature functional channels capable to conduct ionic sodium currents. Furthermore, the voltage-dependence of activation in the two conditions only displayed slight differences, which plays against a dominant role of β2C in the formation of sodium channel complexes and their gating modulation in these cells (Figure 3B).

### β2 promotes filopodia-like processes outgrowth and dendritic branching in developing rat hippocampal neurons

**Figure 4:**
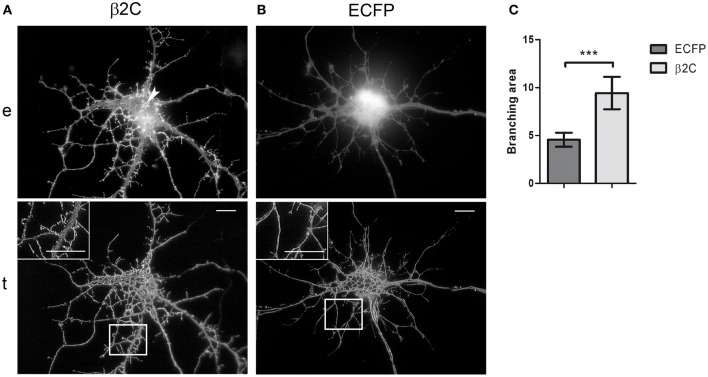
**β2C promotes formation of filopodia-like protrusions and enhances arborization in developing rat hippocampal neurons. (A)** Epifluorescence, *e*, and TIRF, *t*, micrographs of 7 DIV neurons expressing β2C (24 h from transfection). The chimera shows a widespread membrane distribution localizing both in the soma and branches, besides a few small intracellular clusters observable in the perinuclear region (*arrow*). The abundant emergence of filopodia-like protrusions and the expansion of the arborization tree can be noticed in comparison to neurons transfected with ECFP alone **(B)**. Insets in TIRF images show magnifications of white boxes. Scale bars: 10 μm. **(C)** Comparison of branching area index (see Materials and Methods) in neurons transfected with ECFP or β2C. A significant (^***^p = 0.0007) 2-fold increase is observed in β2C (9.4 ± 0.7, *n* = 6) with respect to ECFP expressing neurons (4.5 ± 0.3, *n* = 6).

Taking the cue from the changes in membrane morphology observed in CHO-K1 and to gain insights into the functional role of β2 in neuronal cells, we studied the effect of β2C overexpression in rat hippocampal neurons in culture. Embryonic hippocampal neurons at 18 days of gestation were maintained in culture and transfected at 6 DIV with β2C. At this stage of development, indeed, we usually observed the first appearance of sodium currents in whole-cell electrophysiological recordings, which was signaling the expression onset of mature sodium channels in the plasma membrane. Similarly to CHO-K1 cells, β2C localized mainly in the plasma membrane, despite some perinuclear clustering was also present. Preferential targeting to the cell membrane was confirmed by TIRF microscopy, with the chimera displaying a widespread distribution both in the soma and neuronal branches (Figure 4A). Since from early stages of expression (e.g., 1 or 2 days from transfection), β2C was causing the dynamic appearance of a large number of filopodia-like processes emerging from the branching tree, an effect that was not observed in control neurons transfected with ECFP only (Figure 4B). These newly emerging processes appeared to be similar in morphology and dynamics to those typically observed during development of native hippocampal neurons and that have been suggested as putative precursors of dendritic spines (Ziv and Smith, 1996). Filopodia-like outgrowth led to generation of a highly branched arborization tree. The branching area index (see Materials and Methods) evaluated at the same stage of development, displayed a significant 2-folds increase in β2C neurons (Figure 4C), demonstrating that appearance of β2C-dependent filopodia-like processes was indeed leading to significant expansion of the arborization tree.

**Figure 5:**
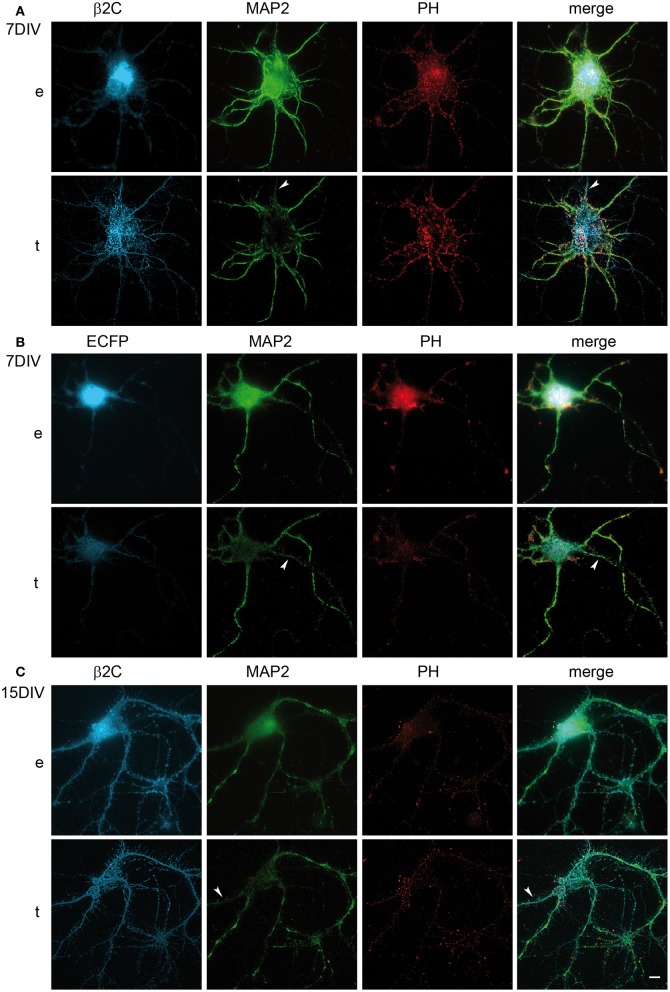
**β2C effect on branching is dendritic specific and is maintained in developing rat hippocampal neurons in culture. (A)** Epifluorescence (*e*) and TIRF (*t*) images of a typical β2C expressing neuron at 7 DIV (24 h from transfection) and stained for MAP2 and Phalloidin (PH) in order to visualize dendritic processes. **(B)** While dendritic arborization is expanded in **(A)**, in control neurons expressing ECFP the effect is not visible. **(C)** β2C expressing neurons at 15 DIV (i.e., 9 days from transfection) maintain enhanced dendritic arborization. **(A–C)**: arrows point to probable axons identified by near absence of MAP2 staining. Scale bars: 10 μm.

**Figure 6:**
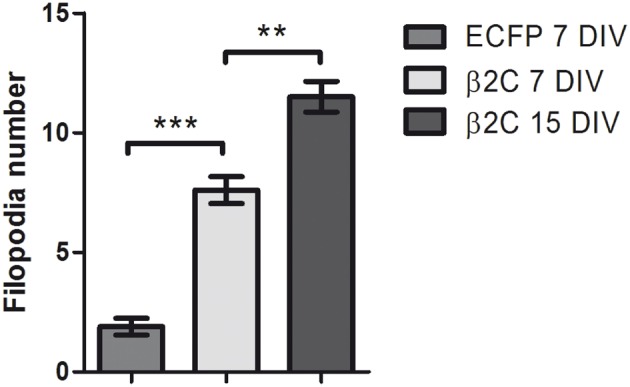
**β2C expression increases the number of dendritic filopodia.** The average count of filopodia-like protrusions in MAP2 stained dendrites is compared in ECFP (1.89 ± 0.35, *n* = 9) and β2C (7.60 ± 0.56, *n* = 10) expressing neurons at 7 DIV. A significant (^***^p < 0.0001) 4-fold increase of filopodia-like processes is observed in β2C expressing cells. Furthermore, the number of processes in β2C expressing neurons at 15 DIV (11.50 ± 0.64, *n* = 4) is significantly higher (^**^p = 0.0019) with respect to 7 DIV neurons. Data expressed as mean ± SEM.

**Figure 7:**
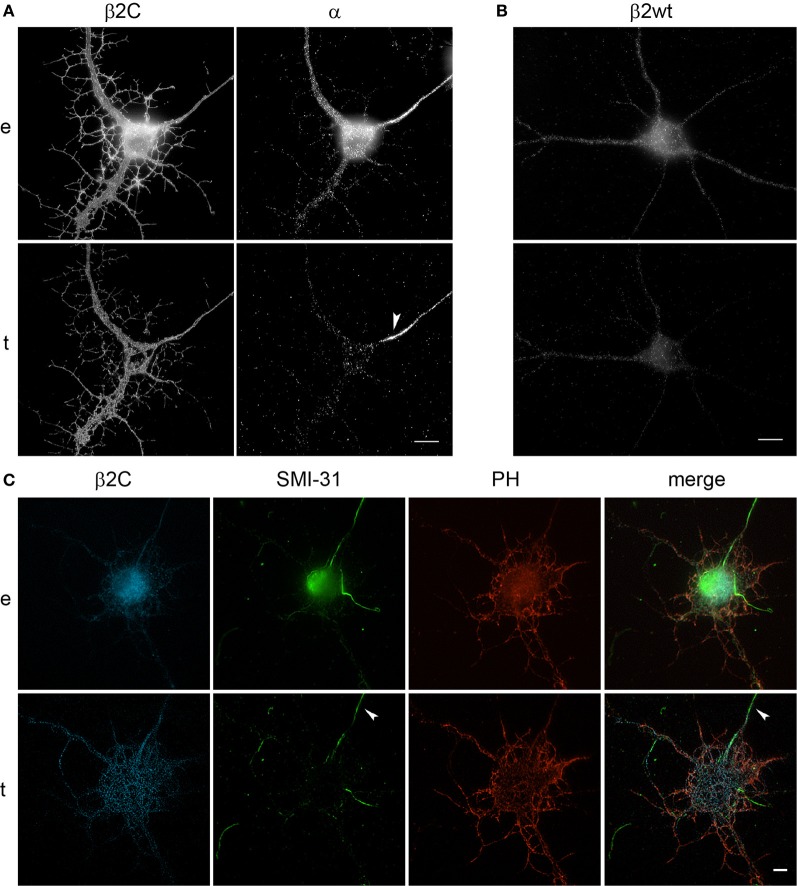
**β2C expression does not affect axonal branching and Na_v_1.2 targeting in developing rat hippocampal neurons. (A)** Epifluorescence (*e*) and TIRF (*t*) images of 7 DIV neurons after 24 h from transfection with β2C and immunolabeled for Na_v_1.2 α subunit. *Left:* β2C channel. *Right*: α channel. Note the staining of the axon by the anti-α antibody (*white arrow*) demonstrating that the typical axonal localization of Na_v_1.2 is not affected by β2C overexpression. Furthermore, as it can be observed in the β2C channel, the branching enhancement observed within the dendritic tree is not present at the axonal level. **(B)** 7 DIV wild-type neuron immunolabeled for the endogenous β2 subunit. β2, as the β2C chimera, shows a widespread distribution in the plasma membrane of both neuronal soma and branches. **(C)** 7 DIV neurons after 24 h from transfection with β2C are stained with SMI-31. Note the absence of filopodia-like structures and enhanced branching in the axon (*arrow*) and the correspondence of β2C and F-actin (PH) signal in dendrites and their protrusions. Scale bars: 10 μm.

Interestingly, we observed that the genesis of filopodia-like structures and enhanced arborization was specific for the dendritic tree. MAP2 and phalloidin staining of β2C expressing 7 DIV neurons evidenced that dendrites undergo extensive branching with respect to ECFP transfected controls (Figures 5A,B). Even after 9 days from β2C transfection (15 DIV), neurons were maintaining an expanded dendritic arborization, thus suggesting that this β2C induced morphological change is not transient but probably conserved during neuronal development (Figure 5C). Image analysis performed on 7 DIV neurons expressing either β2C or ECFP, showed that the average number of emerging filopodia-like processes was about 4-fold higher in β2C neurons than in ECFP controls. In addition, a significant time-dependent increase of filopodia-like processes emerged from comparison between 7DIV and 15DIV β2C transfected neurons (Figure 6). Although MAP2 staining already provided indication that axonal branching is not affected by β2C expression, we specifically investigated neurite morphology by Na_v_1.2 or SMI-31 labeling. Noteworthy, and in contrast to dendrites, axons did not display outgrowth of filopodia-like processes and increased arborization demonstrating that β2C specifically promoted dendritic branching (Figure 7). Interestingly, β2C overexpression did not affect Na_v_1.2 membrane targeting as β2C transfected neurons exhibited unaltered Na_v_1.2 sub-cellular localization, with a preferential targeting of the channel to the axon (Lou et al., 2005) (Figure 7A). Finally, we investigated whether the β2C membrane expression pattern could differ from that one of the native subunit in neurons. As verified by immunolabeling, β2C, and the endogenous β2 exhibited a similar distribution pattern localizing all over the neuronal membrane, including axon and dendrites (Figure 7B).

**Figure 8:**
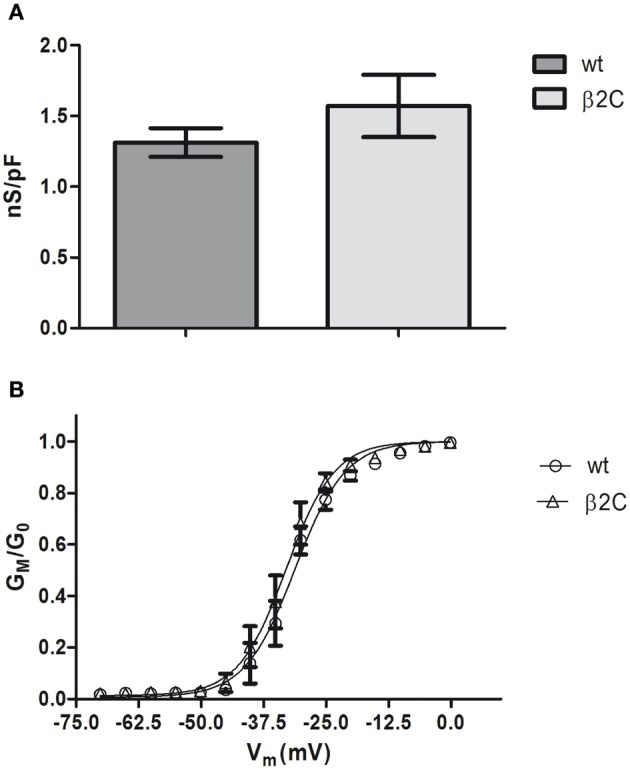
**Sodium-specific conductance and voltage-dependence of activation in β2C transfected rat hippocampal neurons. (A)** Whole-cell peak-specific conductance in rat hippocampal neurons in culture (7 DIV). β2C, β2C expressing neurons at 24 h from transfection; wt, wild-type controls; Voltage protocol, +10 mV test pulse starting from –90 mV holding potential. No significant differences (*p* = 0.3902) of specific conductance were observed in the two conditions (wt = 1.3 ± 0.1 nS/pF, *n* = 8; β2C = 1.6 ± 0.2 nS/pF, *n* = 13). Data are expressed as mean ± SEM. **(B)** Voltage-dependence of activation in wt (*circles*) and β2C (*triangles*) neurons. *G*_*M*_/*G*_0_ indicates the relative conductance (actual conductance/maximum conductance), *V*_*M*_ the intracellular voltage. Fitting by Boltzmann equations show a slight albeit significant shift in the hyperpolarizing direction in β2C expressing neurons (*p* = 0.0064). β2C: *V*_0.5_ = −32.95 ± 0.31 mV, *s* = 4.81 ± 0.26 mV/e-fold conductance change, *n* = 13. wt: *V*_0.5_ = −31.24 ± 0.41 mV, *s* = 5.01 ± 0.35 mV/e-fold conductance change, *n* = 8.

Similarly with what was found in CHO-K1 cells co-transfected with Na_v_1.2 and β2C, peak sodium conductance and activation curve characteristics were not modified by β2C overexpression, as only a slight and non-significant shift in the hyperpolarizing direction was found in the voltage-dependence of activation (Figure 8).

## Discussion

β auxiliary subunits of VGSCs are emerging as multifunctional molecules involved in modulation of sodium channels gating, in their regulation of expression and membrane targeting as well as in cell adhesion-related events such as cell migration or neurite outgrowth and fasciculation. At the root of this is structural versatility, allowing for both formation of channel complexes with α subunits and homo- and heterophilic interactions through extracellular cell adhesion molecule (CAM) domains (Kaczmarek, 2006; Brackenbury and Isom, 2008; Brackenbury et al., 2008a; Patino and Isom, 2010). Among auxiliary subunits, β2 is one of the most studied and evidence has accumulated on its potential for playing heterogeneous functions in the nervous system.

It is likely that apparently unrelated functions of β2 result from differentiated molecular mechanisms that are activated depending on cell type or developmental stage.

One of the most striking effects of β2 expression in host cells is the enhancement of membrane processes and an increase of membrane surface as observed in *Xenopus* oocytes (Isom et al., 1995) and mammalian HEK293 cells (Zimmer et al., 2002). Similarly, the auxiliary subunit could contribute to promote processes outgrowth in neurons, in association, or independently from its α subunit-related functions (Xiao et al., 1999). To investigate this hypothesis, we first characterized sub-cellular localization and effects on generation of membrane processes of a β2 fluorescent chimera, β2C, in CHO-K1 cells, either in the presence or in the absence of Na_v_1.2.

### Effect of β2 on the generation of membrane processes, Na_v_1.2 membrane targeting and gating modulation in CHO-K1 cells

Fluorescence intensities of β2C and FM1-43, a membrane-specific lipophilic dye, were enhanced under TIRF microscopy in correspondence of the cell periphery and of lamellipodia- and filopodia-like membrane protrusions (Figures 1A,B). While a higher FM1-43 signal suggested tighter adhesion in those regions that are involved in cell spreading and migration (Mattila and Lappalainen, 2008), the option of a concomitant β2C protein accumulation had also to be considered. To investigate chimera accumulation, FM1-43 fluorescence was subtracted from the β2C signal by an intensity difference analysis, as described in Materials and Methods. As a result, β2C appeared to be expressed at higher levels at the cell periphery and within membrane processes (Figure 1B). A statistical analysis of β2C signal intensity along segments crossing the cell's edge confirmed the preferential localization of the subunit at the cell border, even outside lamellipodia-like structures that were excluded from the analysis (Figures 1C,D). Considering also that β2C expressing cells were displaying a clear increase of lamellipodia- and filopodia-like protrusions and that the subunit had a localization pattern similar to actin filaments (Figure 1A), the results were suggesting that β2C was promoting membrane adhesion and spreading in CHO-K1 cells. Cell spreading and migration are generally thought to be mediated by filopodia and lamellipodia enriched with adhesion molecules for the extracellular matrix such as cadherins and integrins (Galbraith et al., 2007; Chioni et al., 2009; Parsons et al., 2010). β2 could act in a similar manner, first by probing the extracellular matrix by its CAM domain, then activating intracellular signals leading to strengthened actin polymerization and processes formation to drive cell motion. At partial support to this hypothesis, it was previously shown that blockade of γ-secretase cleavage of β2 C-terminal fragment inhibits cell migration (Kim et al., 2005), which implies that β2 is indeed capable of signaling formation of membrane processes.

### Mutual intracellular retention of Na_v_1.2 and β2 in CHO-K1 cells

Na_v_1.2 is one of the most important α subunit isoforms in the mammalian central nervous system (Catterall, 2000; Lai and Jan, 2006) and is highly represented in the hippocampus with a preferential localization in unmyelinated axons (Westenbroek et al., 1989; Gong et al., 1999). Since Na_v_1.2 has been shown to form complexes with β2 (Schmidt and Catterall, 1987; Gong et al., 1999), we investigated the effect on membrane targeting and sodium current properties of this channel in β2C co-expressing CHO-K1 cells. A significant retention of Na_v_1.2 and β2C within endomembranes was observed, reminding the distribution described in developing rat brain neurons (Schmidt et al., 1985; Gong et al., 1999) (Figure 2A). Na_v_1.2 targeted more efficiently to the plasma membrane when expressed alone, despite a relevant intracellular retention as observed for Na_v_1.5 in other cell lines (Zimmer et al., 2002) (Figures 2B,C). Interestingly, measurements of whole-cell sodium conductance showed a similar degree of expression of functional channels under the two conditions (Figure 3A) and, at the gating level, we did not observe major differences in the voltage-dependence of activation (Figure 3B). Taken together, these results suggested that β2C/Na_v_1.2 co-expression caused intracellular retention of both subunits but without significantly altering the targeting of functional channels to the membrane. Thus, we may hypothesize that, in CHO-K1 cells, conducting channels are likely formed by a subpopulation of α subunits that are directed to the membrane independently from β2C association. On the other hand, hindering of β2C targeting to the plasma membrane by Na_v_1.2 markedly reduced the appearance of cell processes (Figure 2). We concluded that β2C capability to promote processes outgrowth was directly related to its targeting to the cell membrane.

### β2 enhances dendritic filopodia and arborization in developing rat hippocampal neurons

To gain insights into the subunit's role in neurons, we overexpressed β2C in embryonic rat hippocampal neurons developing in culture. While β2 expression occurs in the earliest phase of neurogenesis in the rat brain and is greatly increased concomitantly with axon extension and synaptogenesis (Isom et al., 1995), its association to Na_v_1.2 appears only at later stages (Gong et al., 1999). Thus, it is possible that during an early developmental time window β2 contributes mainly to neuronal wiring rather than sodium channel localization and gating modulation. Thus, we chose to transfect exogenous β2C in neurons at 6 DIV when dendritic and axonal outgrowth and branching are actively ongoing. As at the same stage we observed the onset of sodium current appearance in our cultures, we concomitantly assessed the effects of β2C on sodium channels targeting and gating. Overexpression of β2C led to a clear increase of filopodia-like protrusions in the dendritic tree with respect to ECFP transfected controls (Figures 4A,B). Interestingly, their morphology and dynamic appearance and disappearance closely resembled those of native dendritic filopodia (Ziv and Smith, 1996), which are thought to lead to dendrite formation (Heiman and Shaham, 2010). The number of filopodia-like processes was displaying a 4-fold increase with respect to controls (Figure 6). The β2C-mediated induction of newly emerging filopodia led to an expanded arborization with a 2-fold increase of branching area (Figure 4C). The effect was dendrite-specific and the axon did not display either filopodia-like protrusions or branching enhancement (Figures 5, 7). Furthermore, the expansion of the dendritic tree induced by β2C appeared to be potentially relevant for development as it was maintained even after 9 days from transfection (Figure 5C). Also the emergence of newly formed filopodia-like structures was increasing over time as observed in 15 DIV β2C expressing neurons with respect to 7DIV cells (Figure 6). In order to exclude that the aforementioned effects were produced by anomalous sub-cellular distribution of the chimera, the sub-cellular localization of β2C was compared to that one of the native β2 subunit. In both cases the auxiliary subunit displayed a widespread distribution over the whole neuronal membrane, including soma and branches (Figure 7B). Interestingly, β2C overexpression did not alter the typical Na_v_1.2 targeting to the axon (Figure 7A). This observation was in agreement with previous work suggesting that a targeting sequence within linker II–III of the pore-forming subunit is sufficient to localize and segregate Na_v_1.2 channels in the axon initial segment (Garrido et al., 2003). Also, β2C overexpression did not affect sodium conductance levels and voltage-dependence of activation (Figure 8), suggesting that either α subunit alone or in association with endogenous β2 is forming conductive sodium channels in these neurons.

In conclusion, β2 overexpression significantly promoted the emergence of dendritic filopodia and the expansion of the dendritic tree in developing neurons while it had negligible effects on sodium channels targeting and gating modulation. The specificity of β2 action for the dendritic tree is suggestive for a role of the subunit in the modulation of neuronal wiring *in vivo*. Scn2b null mice showed susceptibility to epileptic seizures (Chen et al., 2002) where abnormal dendritic development could be involved in addition to the observed negative shift in the voltage-dependence of inactivation. However, no broad impairment of nervous system function was evidenced in these mice. Despite that, given its high physiological expression levels throughout the central nervous system, β2 is likely to contribute to normal development. Compensatory mechanisms may exist that are activated in Scn2b null mice, such as expression of the structurally related β4 subunit that has been also found to promote dendritic outgrowth (Miyazaki et al., 2007). Interesting is the analogy with the observed stimulation of neurite extension and fasciculation by β1 in cerebellar granule cells (Davis et al., 2004; Brackenbury et al., 2008a), which was prevented by β2 co-expression. Although further work will be required to investigate the hypothesis, we may speculate that the two subunits contribute to differentially promote axonal and dendritic wiring during early neuronal development, while association with α may downplay this function by subtracting the auxiliary subunits from their free pool at later time points.

### Conflict of interest statement

The authors declare that the research was conducted in the absence of any commercial or financial relationships that could be construed as a potential conflict of interest.
